# The Part Played by the Nose and the Anterior Respiratory Passages in Respiration

**Published:** 1888-02

**Authors:** 


### The Part Played by the Nose and the Anterior Respiratory Passages in Respiration.

R. Kayser reports in *Pfluger's Archiv* some investigations upon the part played by the nasal cavities in the warming and vaporization of the inspired air. In these he differs from the results obtained by Aschenbrandt. From the *Centralblatt f. d. Med. Wissensch.*, November 5, 1887, we learn that Kayser regards as incorrect the assertion that a vital role attaches to nasal respirations, which can not be accomplished by mouth and throat breathing. By means of a series of experiments, similar to those employed by Aschenbrandt, he established the most essential results of the latter's researches. He finds that air passing through both nasal cavities (five liters in 30 seconds), when the temperature of the outside air is from 50°–54° F., is warmed to about 88° F., and is completely saturated with vapor; and this occured although he was careful to hermetically seal the nasal cavities by firm pressure of the palate against the posterior nasal fossæ.

This result is not changed if the rapidity of the current of air is increased upon both sides. If the temperature of the outside air is lower, the degree of warming obtained is less, but is always decided; air at a temperature of 32°-39° F. is warmed to a temperature of 81.5° F. If the experiment is so conducted that the current of air, instead of passing through the nose, passes through the mouth and throat, then the degree of warming obtained is only slightly less, while the saturation with moisture is just as complete. The author also thinks that the deeper respiratory passages must play a role similar to that of the nose or mouth and throat, if the air which reaches them is not already warm and moist. But in normal nasal breathing, the greater part of the work devolves upon the nasal cavities.

The opinion of Aschenbrandt with reference to the purification of the air from dust as it passes through the nose, Kayser does not find verified in his own experiments. He thinks, moreover, that a certain quantity of particles of dust must penetrate even into the lungs.

